# The Effect of Tracheal Intubation-Induced Autonomic Response on Photoplethysmography

**DOI:** 10.1155/2017/7646541

**Published:** 2017-04-02

**Authors:** Pekka Talke

**Affiliations:** University of California San Francisco, 500 Parnassus Avenue, San Francisco, CA 94143, USA

## Abstract

*Introduction*. Intraoperative stress responses and postoperative pain can be monitored using photoplethysmography (PPG). PPG signal has two components, AC and DC. Effects of noxious stimuli-induced stress responses have not been studied on the DC component of PPG. The aim of this study was to investigate the effect of a known noxious stimulus (endotracheal intubation) on both the AC and DC components of PPG.* Methods*. 15 surgical patients having general anesthesia were enrolled into this clinical study. PPG was recorded electronically from a pulse oximeter. Maximum changes in the AC and DC components of the PPG and pulse rate were determined in response to endotracheal intubation from high frequency (62.5 Hz) PPG recordings.* Results*. Endotracheal intubation-induced autonomic stress response resulted in a significant decrease in the AC component of the PPG and an increase in pulse rate in every subject (*p* < 0.05 for all). The decrease in the AC component of the PPG was 50 ± 12% (*p* < 0.05) and the increase in pulse rate was 26 ± 10 bpm (*p* < 0.05). The response of the DC component was variable (*p* = NS).* Conclusion*. Endotracheal intubation-induced stress response resulted in a significant and consistent change in the AC, but not the DC component of the PPG. This trial is registered with ClinicalTrials.gov Identifier NCT03032939.

## 1. Introduction

**Figure 1:**
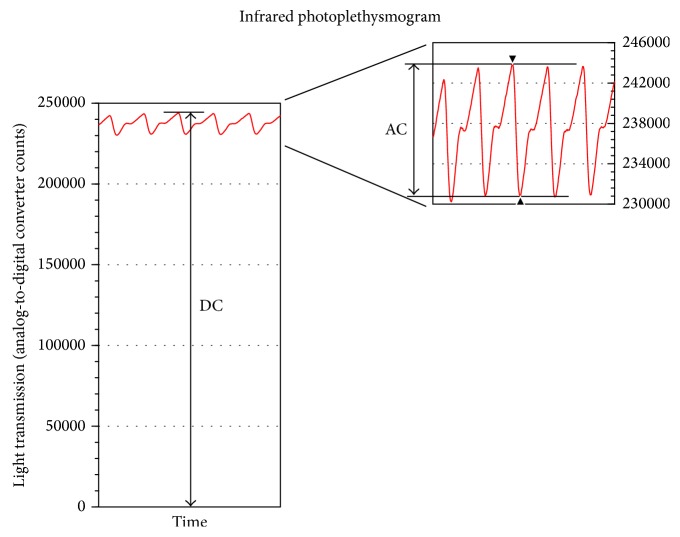
Illustration of photoplethysmogram DC and AC components. The DC value corresponds to the smallest blood volume in the finger (end diastole), which corresponds to the highest light transmission value of each pulse through the finger. The AC values correspond to the difference between the highest (end diastole) and lowest (end systole) light transmission values of each pulse representing the pulse added volume of blood in the finger. Units for the photoplethysmogram DC and AC components are analog-to-digital converter counts that are proportional to the light transmission in Watts.

Photoplethysmography (PPG) derived variables have been used to quantitate nociception-induced autonomic responses such as changes in vasomotor tone and heart rate [1–9]. Because of it is noninvasive nature, PPG is a practical tool in pain research. PPG uses infrared light transmission to quantitate vasomotor tone (vasoconstriction and vasodilation) by measuring changes in tissue volume [10–14]. The light transmission PPG signal is composed of two components, commonly referred to as AC and DC (Figure 1). The AC component of the signal is mostly a result of the pulsating portion of arterial blood. The DC component of the PPG signal is due to light transmission through tissues with minimal volume changes, venous blood, and the nonpulsating portion of arterial blood. Thus, changes in vasomotor tone can have an effect on both the AC and DC components of the PPG signal.

Most previous clinical studies that have used PPG-derived variables to evaluate the effects of nociception on autonomic responses have used only the AC component of PPG [1–4, 6–9, 15–17]. Since peripheral vasoconstriction may have an effect on both the AC and DC components of PPG, and the DC component may contain valuable information that has not been intensively explored, we hypothesized that autonomic responses induced by a noxious stimulus would decrease the AC component (reduced arterial pulsation) and increase the DC component (reduced venous and nonpulsatile arterial blood volume) of the PPG. Thus, the aim of this study was to investigate the effect of a noxious stimulus on both the AC and DC components of the PPG. We used tracheal intubation as a standard noxious stimulus to elicit an autonomic stress response.

## 2. Materials and Methods

With approval of the Institutional Review Board, and written informed consent, we studied 15 patients who were scheduled for elective surgery under general anesthesia. We excluded individuals who had a history of cardiac, vascular, hepatic, or renal disease; those less than 18 years old; and those weighing more than 130% of normal body mass index.

On the day of study, a catheter was inserted into a vein of the hand to permit administration of intravenous fluids and medications. During the study, patients were in supine position, covered with a light blanket on the operating room table. Prior to induction of anesthesia, standard anesthesia monitors (5-lead ECG, noninvasive blood pressure cuff, and a pulse oximeter probe) were applied.

Patients received midazolam and fentanyl premedication. Patients breathed 100% oxygen before and during induction of general anesthesia with intravenous propofol. Administration of rocuronium facilitated tracheal intubation. Timing of the administration of propofol and rocuronium, beginning of laryngoscopy, and completion of endotracheal intubation were marked electronically using an automated data-acquisition system (see below). To minimize movement related artifact on the PPG signal, endotracheal intubation was performed 3 min after administration of rocuronium (0.6 mg/kg). Neuromuscular transmission monitors were not used as they can have a detrimental effect on the PPG signal. Endotracheal intubation was performed by the author using a size-3 Macintosh laryngoscope blade. No additional medications were administered till end of data collection.

Infrared light transmitted through a fingertip (PPG) was measured by a Masimo Radical-7 pulse oximeter (Masimo Corp., Irvine, CA, Masimo SET software version 7.0.3.3), for which we placed an adhesive LNCS Adtx sensor (Masimo Corp., Irvine, CA) on the ring finger contralateral to the arm with the blood pressure cuff. PPG data were recorded by an automated data-acquisition system (see below) starting before induction of anesthesia till 2 min after endotracheal intubation.

The pulse oximeter consists of two parts, a sensor and a monitor. The sensor, which is applied to the tip of a finger, contains a low-voltage, low-intensity, light-emitting diode that emits infrared light (approximately 910 nm). A portion of this light is transmitted through the finger. A detector photodiode in the sensor generates an electrical current proportional to the amount of light received. This electrical current is converted to analog-to-digital converter units and low-pass-filtered (10 Hz). Data on electrical current thus generated were transmitted to a computer, sampled at 62.5 Hz, and recorded with no further signal processing. These data were recorded using a Pulse Ox Automated Data Collection (ADC) software (Masimo Corp., Irvine, CA, ADC v3.1.1.0), that collects signals from pulse oximeter probes for engineering purposes. The same software was used to collect data on timing of the events.

### 2.1. Statistics

Sample size analysis was not performed, as there were no available preliminary data. PPG data were analyzed offline. For each arterial pulse, maximum and minimum light transmittances were identified from the high frequency (62.5 Hz) PPG data. Then the AC and DC component values of the PPG were determined for each pulse. AC and DC data were further used to calculate a derived variable, perfusion index (PI), which is defined as PI = AC/DC *∗* 100%. In addition, pulse rate (PR) was calculated from beat-to-beat time interval data.

For analysis, we included data from 5 heartbeats immediately before and for 55 heartbeats immediately following the beginning of the intubation-induced stress response. Baseline values for all continuous variables (AC, DC, PI, and PR) were defined as the median value of the 5 heartbeats immediately before the stress response. Repeated-measures analysis of variance followed by Dunnett's post hoc test was used to determine the effect of endotracheal intubation on AC, DC, PI, and PR. Data are reported as mean ± SD, with *p* < 0.05 signifying statistical significance.

## 3. Results

**Figure 2:**
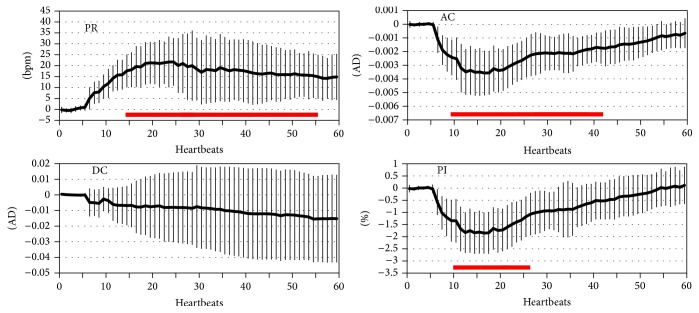
Change (absolute values) in pulse rate (PR) and photoplethysmogram component (AC, DC, and PI) data from baseline values. Data are mean (thick line) ± SD over 60 heartbeats. Data is illustrated for 5 heartbeats before and 55 heartbeats after the beginning of the response. The red horizontal bars illustrate values that are significantly (*p* < 0.05) different from baseline values. Units for AC and DC are analog-to-digital converter counts (AD).

Out of 15 subjects, 5 subjects were excluded from analysis for technical (3) and movement related artifact (2) reasons. Demographic and medication data of the remaining 10 subjects are shown in Table 1. Average time from beginning of laryngoscopy to end of intubation was 9 ± 2 seconds. AC and PI decreased and PR increased in response to endotracheal intubation in all subjects (*p* < 0.05 for all) (Figure 2). DC increased in 3 subjects and decreased in 7 subjects in response to endotracheal intubation (*p* = NS) (Figure 2).

In relation to baseline values, maximum decreases in AC and PI were 50 ± 12% (*p* < 0.05) and 48 ± 14% (*p* < 0.05), respectively. On average, the maximum decrease in AC and PI was reached 12 heartbeats after the beginning of the stress response. Maximum increase in PR was 26 ± 10 bpm (*p* < 0.05). The maximum increase in PR was reached on average 22 heartbeats after the beginning of the stress response.

## 4. Discussion 

Our study is the first to evaluate the effect of a noxious stimulus (tracheal intubation) on both the AC and DC components of the PPG. Our results show that endotracheal intubation-induced stress response mediates peripheral vasoconstriction that results in a decrease in the AC component of the PPG and PI. The decrease in PI was secondary to the reduction in the AC component of the PPG. Contrary to our hypothesis, we did not find a consistent or significant increase in the DC component of the PPG.

PPG-derived variables can be used to measure nociception-induced autonomic nervous system responses [2, 3]. Hamunen et al. demonstrated that several PPG-derived variables could be used to measure autonomic nervous system activation in awake healthy volunteers when using noxious thermal stimuli [2]. PPG-derived variables have also been used in anesthetized patients to measure autonomic nervous system activation. One such variable is Surgical Stress Index (SSI), which uses PPG and pulse-to-pulse interval data to evaluate nociception/antinociception balance during general anesthesia [3]. Several studies have demonstrated that SSI reacts to surgical stress and that increasing doses of opioids and anesthetics can attenuate these responses [1, 8, 9, 18].

Most of the previous studies utilized only the AC component of PPG [1–4, 6–9, 15–17]. However, the DC component of PPG is also known to change in response to vasoconstriction and vasodilation. For example, elimination of autonomic nervous system activity using an axillary nerve conduction block results in a decrease in the DC component of PPG (vasodilation) while surprisingly having no effect on the AC component [19]. Thus, understanding the effects of various physiological events on both the AC and DC components of PPG is important in optimizing the amount of physiological information that could be derived from the PPG signal [12–14, 19].

We used endotracheal intubation as an established, distinct, noxious stimulus because it induces an extensively studied and well-characterized stress response [20–26]. Tracheal intubation results in an increase in arterial blood pressure, heart rate, and plasma catecholamine levels, all of which reach a peak within a minute after the stimulus [20–22, 24–26]. Although this stress response may be attenuated by opioids and anesthetics [8, 16, 20, 25, 27, 28], it will not be eliminated with clinically relevant anesthetic doses [18, 25, 27, 28]. Our anesthetic regimen achieved the goals of this study; all of our patients had a significant stimulus induced stress response as demonstrated by the decreases in the AC component of the PPG and increases in PR.

The fact that so many patients were excluded for poor data quality deserves comment. PPG is extremely sensitive to even the slightest movement, creating unacceptably large artifacts. We specifically chose not to use neuromuscular transmission monitors, because use of neuromuscular monitors is known to induce an electrical stimulus that results in peripheral vasoconstriction, thus, interfering with our data [3, 4, 7]. Despite the 3 min time interval between administration of rocuronium and endotracheal intubation, two patients moved their arms during intubation. This was due to lack of complete neuromuscular blockade before endotracheal intubation.

Variability in the response of the DC component of the PPG was unexpected and deserves comment. Autonomic stress response mediates peripheral vasoconstriction as seen in the consistent decrease in the AC component of PPG. Theoretically the decrease in arterial size should have increased light transmission and thus resulted in an increase in the DC component of PPG. Although highly speculative, the unexpected decrease in the DC component of the PPG in 7 subjects could have been due to reduced venous return (increased venous volume in finger) secondary to poor relaxation and resulting diaphragmatic activity. Consistent with this theory, breath holding (valsalva) in awake subjects will result in a decrease the DC component of PPG.

Tracheal intubation is a brief (seconds), intense stimulus. The resulting peripheral vasoconstrictive response was also surprisingly brief. AC values decreased rapidly and reached minimum values, on the average, within 12 heartbeats after the beginning of the response, and returned to baseline values approximately 30–40 heartbeats later. In contrast, the PR response peaked later and was significantly longer in duration. We speculate that the PR response is prolonged in part by increased levels of circulating catecholamines whereas the peripheral vasoconstriction that we observed is mediated mainly by brief sympathetic nervous system activation.

In conclusion, the results of this study show that tracheal intubation-induced noxious stimulus resulted in a significant change in the AC but not the DC component of the PPG. The decrease in PI was secondary to the changes in the AC component of the PPG. The reason for the variability in the response of the DC component of the PPG was unexpected and remains unknown.

## Figures and Tables

**Table 1:** Demographics, baseline hemodynamic values, and induction agents.

Variable	(*n* = 10)
Age (years)	46 ± 15
Gender (M/F)	5/5
Weight (kg)	81 ± 16
ASA (1/2/3)	3/4/3

SBP (mmHg)	131 ± 25
DBP (mmHg)	76 ± 12
PR (bpm)	89 ± 23

Midazolam (mg)	1.3 ± 0.5
Fentanyl (ug/kg)	0.9 ± 0.3
Propofol (mg/kg)	1.9 ± 0.6
Rocuronium (mg/kg)	0.6 ± 0.1

ASA = American Society of Anesthesiologists classification; SBP = systolic blood pressure; DBP = diastolic blood pressure; PR = pulse rate.

## References

[1] Ahonen J., Jokela R., Uutela K., Huiku M. (2007). Surgical stress index reflects surgical stress in gynaecological laparoscopic day-case surgery. *British Journal of Anaesthesia*.

[2] Hamunen K., Kontinen V., Hakala E., Talke P., Paloheimo M., Kalso E. (2012). Effect of pain on autonomic nervous system indices derived from photoplethysmography in healthy volunteers. *British Journal of Anaesthesia*.

[3] Huiku M., Uutela K., van Gils M. (2007). Assessment of surgical stress during general anaesthesia. *British Journal of Anaesthesia*.

[4] Rantanen M., Yli-Hankala A., van Gils M. (2006). Novel multiparameter approach for measurement of nociception at skin incision during general anaesthesia. *British Journal of Anaesthesia*.

[5] Rantanen M., Yppärilä-Wolters H., van Gils M. (2007). Tetanic stimulus of ulnar nerve as a predictor of heart rate response to skin incision in propofol-remifentanil anaesthesia. *British Journal of Anaesthesia*.

[6] Seitsonen E. R. J., Korhonen I. K. J., Van Gils M. J. (2005). EEG spectral entropy, heart rate, photoplethysmography and motor responses to skin incision during sevoflurane anaesthesia. *Acta Anaesthesiologica Scandinavica*.

[7] Luginbühl M., Rüfenacht M., Korhonen I., Gils M., Jakob S., Petersen-Felix S. (2006). Stimulation induced variability of pulse plethysmography does not discriminate responsiveness to intubation. *British Journal of Anaesthesia*.

[8] Struys M. M. R. F., Vanpeteghem C., Huiku M., Uutela K., Blyaert N. B. K., Mortier E. P. (2007). Changes in a surgical stress index in response to standardized pain stimuli during propofol-remifentanil infusion. *British Journal of Anaesthesia*.

[9] Wennervirta J., Hynynen M., Koivusalo A.-M., Uutela K., Huiku M., Vakkuri A. (2008). Surgical stress index as a measure of nociception/antinociception balance during general anesthesia. *Acta Anaesthesiologica Scandinavica*.

[10] Allen J. (2007). Photoplethysmography and its application in clinical physiological measurement. *Physiological Measurement*.

[11] Nitzan M., Babchenko A., Khanokh B., Landau D. (1998). The variability of the photoplethysmographic signal—a potential method for the evaluation of the autonomic nervous system. *Physiological Measurement*.

[12] Talke P., Stapelfeldt C., Lobo E., Brown R., Scheinin M., Snapir A. (2005). Effect of *α*2B-adrenoceptor polymorphism on peripheral vasoconstriction in healthy volunteers. *Anesthesiology*.

[13] Talke P. O., Lobo E. P., Brown R., Richardson C. A. (2001). Clonidine-induced vasoconstriction in awake volunteers. *Anesthesia and Analgesia*.

[14] Talke P., Lobo E., Brown R. (2003). Systemically administered *α*2-agonist-induced peripheral vasoconstriction in humans. *Anesthesiology*.

[15] Awad A. A., Ghobashy M. A. M., Ouda W., Stout R. G., Silverman D. G., Shelley K. H. (2001). Different responses of ear and finger pulse oximeter wave form to cold pressor test. *Anesthesia and Analgesia*.

[16] Luginbühl M., Reichlin F., Sigurdsson G. H., Zbinden A. M., Petersen-Felix S. (2002). Prediction of the haemodynamic response to tracheal intubation: comparison of laser-Doppler skin vasomotor reflex and pulsed wave reflex. *British Journal of Anaesthesia*.

[17] Zhang L., Xu L., Zhu J. (2014). To clarify features of photoplethysmography in monitoring balanced anesthesia, compared with cerebral state index. *Medical Science Monitor*.

[18] Mustola S., Parkkari T., Uutela K., Huiku M., Kymäläinen M., Toivonen J. (2010). Performance of surgical stress index during sevoflurane-fentanyl and isoflurane-fentanyl anesthesia. *Anesthesiology Research and Practice*.

[19] Talke P., Snapir A., Huiku M. (2011). The effects of sympathectomy on finger photoplethysmography and temperature measurements in healthy subjects. *Anesthesia and Analgesia*.

[20] Albertin A., Casati A., Federica L. (2005). The effect-site concentration of remifentanil blunting cardiovascular responses to tracheal intubation and skin incision during bispectral index-guided propofol anesthesia. *Anesthesia and Analgesia*.

[21] Barak M., Ziser A., Greenberg A., Lischinsky S., Rosenberg B. (2003). Hemodynamic and catecholamine response to tracheal intubation: direct laryngoscopy compared with fiberoptic intubation. *Journal of Clinical Anesthesia*.

[22] Derbyshire D. R., Chmielewski A., Fell D., Vater M., Achola K., Smith G. (1983). Plasma catecholamine responses to tracheal intubation. *British Journal of Anaesthesia*.

[23] Durrani M., Barwise J. A., Johnson R. F., Kambam J. R., Janicki P. K. (2000). Intravenous chloroprocaine attenuates hemodynamic changes associated with direct laryngoscopy and tracheal intubation. *Anesthesia and Analgesia*.

[24] Haidry M. A., Khan F. A. (2013). Comparison of hemodynamic response to tracheal intubation with Macintosh and McCoy laryngoscopes. *Journal of Anaesthesiology Clinical Pharmacology*.

[25] Hoda A., Khan F. A. (2011). Effect of one minimum alveolar concentration sevoflurane with and without fentanyl on hemodynamic response to laryngoscopy and tracheal intubation. *Journal of Anaesthesiology Clinical Pharmacology*.

[26] Shimoda O., Ikuta Y., Sakamoto M., Terasaki H. (1998). Skin vasomotor reflex predicts circulatory responses to laryngoscopy intubation. *Anesthesiology*.

[27] Hassani V., Movassaghi G., Goodarzi V., Safari S. (2013). Comparison of fentanyl and fentanyl plus lidocaine on attenuation of hemodynamic responses to tracheal intubation in controlled hypertensive patients undergoing general anesthesia. *Anesthesiology and Pain Medicine*.

[28] Ko S.-H., Kim D.-C., Han Y.-J., Song H.-S. (1998). Small-dose fentanyl: optimal time of injection for blunting the circulatory responses to tracheal intubation. *Anesthesia and Analgesia*.

